# Enhancement of Methacholine-Evoked Tracheal Contraction Induced by Bacterial Lipopolysaccharides Depends on Epithelium and Tumor Necrosis Factor

**DOI:** 10.1155/2012/494085

**Published:** 2012-03-11

**Authors:** T. Secher, F. Rodrigues Coelho, N. Noulin, A. Lino dos Santos Franco, V. Quesniaux, J. Lignon, J. Mitchell, R. Moser, E. Gomes, L. Mirotti, W. Tavares-de-Lima, B. Ryffel, B. Boris Vargaftig, M. Russo

**Affiliations:** ^1^Laboratory Molecular Immunology and Embryology, University of Orleans and CNRS, Orleans, France; ^2^Department of Immunology, Institute of Biomedical Sciences, University of Sao Paulo, Avenue Prof. Lineu Prestes, 1730, 05508-000 São Paulo, SP, Brazil; ^3^Cardiothoracic Pharmacology, Cardiothoracic and Stem Cell Pharmacology, National Heart and Lung Institute, Imperial College of London, London SW7 2AZ, UK; ^4^Institute for Clinical and Biomedical Research Thurgau, Lauchefeld 31, CH-9548 Matzingen, Switzerland; ^5^Institute of Infectious Disease and Molecular Medicine (IIDMM), University of Cape Town, Cape Town, South Africa

## Abstract

Inhaled bacterial lipopolysaccharides (LPSs) induce an acute tumour necrosis factor-alpha (TNF-**α**-) dependent inflammatory response in the murine airways mediated by Toll-like receptor 4 (TLR4) via the myeloid differentiation MyD88 adaptor protein pathway. However, the contractile response of the bronchial smooth muscle and the role of endogenous TNF**α** in this process have been elusive. We determined the *in vivo* respiratory pattern of C57BL/6 mice after intranasal LPS administration with or without the presence of increasing doses of methacholine (MCh). We found that LPS administration altered the basal and MCh-evoked respiratory pattern that peaked at 90 min and decreased thereafter in the next 48 h, reaching basal levels 7 days later. We investigated in controlled *ex vivo* condition the isometric contraction of isolated tracheal rings in response to MCh cholinergic stimulation. We observed that preincubation of the tracheal rings with LPS for 90 min enhanced the subsequent MCh-induced contractile response (hyperreactivity), which was prevented by prior neutralization of TNF**α** with a specific antibody. Furthermore, hyperreactivity induced by LPS depended on an intact epithelium, whereas hyperreactivity induced by TNF**α** was well maintained in the absence of epithelium. Finally, the enhanced contractile response to MCh induced by LPS when compared with control mice was not observed in tracheal rings from TLR4- or TNF- or TNF-receptor-deficient mice. We conclude that bacterial endotoxin-mediated hyperreactivity of isolated tracheal rings to MCh depends upon TLR4 integrity that signals the activation of epithelium, which release endogenous TNF**α**.

## 1. Introduction

Airway inflammation due to environmental agents, including endotoxin or its identified component lipopolysaccharide (LPS), plays an important role in the progression of chronic respiratory diseases [1–5]. Experimental inhalation of LPS in mice induces local TNF*α* production, neutrophil recruitment, and injury to alveolar epithelium and endothelium, with protein leakage into the alveolar space [6, 7], followed by bronchoconstriction and hyperresponsiveness in response to methacholine [8, 9].

Toll-like receptor 4 (TLR4) and CD14 play a critical role in the lung response to systemic LPS administration [10–12], although other genes may be also involved in the biological responses to inhaled LPS [13]. We have shown that the level of TLR4 expression determines the extent of the acute lung response to inhaled LPS [14]. However, neutrophil recruitment from the pulmonary microvasculature to the lung tissue and bronchoalveolar space, following the aerogenic exposure to LPS, is MAPK dependent but TNF*α* independent [8]. 

The effects of systemic administration of LPS in the lungs differ from those of its aerogenic administration, since LPS administration is not followed by direct bronchoconstriction, but by bronchial hyperresponsiveness to serotonin in guinea pigs [15] and to MCh in mice [8], and this is suppressed by glucocorticosteroids. By contrast, even though glucocorticosteroids suppress TNF*α* production, they fail to interfere with lung neutrophil recruitment or with direct bronchoconstriction due to aerogenic LPS [12]. This suggests that different mechanisms account for LPS effects, depending on the route of administration and on the first cells activated. One of the potential targets of LPS is the epithelium lining the interface between the air space and the mucosal tissue. Experimental evidence suggests that the airway epithelium releases contractile and relaxing factors [16], although a direct effect of LPS on airway smooth muscle cannot be excluded [16]. 

In order to study the effects of LPS on airways, mice received aerogenic LPS and the respiratory parameters obtained by noninvasive and invasive methods were determined before and after inhaled methacholine. In order to circumvent the controversy of noninvasive versus invasive, and the complex *in vivo* interactions such as the effects of vagus nerve modulation and interference of neutrophil recruitment into the lungs with smooth muscle contractility, we performed experiments *in vitro* with isolated tracheal segments. We studied the role of epithelium on cholinergic responsiveness of the airway, the contractile response of tracheal segments to MCh when preincubated with LPS. Furthermore, the changes in tracheal response to MCh induced by TNF*α*, as the major potential mediator released by LPS, were also investigated. 

## 2. Material and Methods

### 2.1. Animals

Male (8–12 weeks old) mice were kept in sterile isolated ventilated cages. C57BL/6, TNF^−/−^[17], TNFR1/R2^−/−^[18, 19], and naturally TLR4-deficient mice [20] were backcrossed 10 times on the C57BL/6 genetic background and bred under specific-pathogen-free conditions at the CNRS, Orleans, France. All experiments were approved by the local ethical Committee, and in accordance with the European Council Guidelines for the Care and Use of Laboratory animals. 

### 2.2. Endotoxin (LPS) Administration

Twenty micrograms of LPS from *Escherichia coli* (serotype 055: B5, Sigma St Louis, MO, USA) were given by the aerogenic route using nasal instillation in a volume of 30 mL under light ketamine-xylasine anesthesia, and pulmonary functions were determined by barometric noninvasive and invasive methods.

### 2.3. Determination of Airway Responsiveness by Noninvasive and Invasive Barometric Plethysmography

Noninvasive analyses of respiratory parameters were determined by barometric whole-body plethysmography (Buxco Electronics Inc. Wilmington, NC, USA) at 1.5, 24, 48, and 168 h after LPS or PBS administration in conscious unrestrained mice as previously described [21, 22]. After 24 h of LPS administration, respiratory parameters were determined before and after increasing doses of methacholine. Signals were analyzed using BioSystem XA software to derive whole-body flow parameters including respiratory frequency, tidal volume, minute ventilation, peak inspiratory flow, peak expiratory flow, and enhanced pause (Penh). Penh is a dimensionless value that takes into account box pressure recorded during inspiration and expiration and the timing comparison of early and late expiration, which was used to define the respiratory breathing pattern.

Invasive analysis of lung function was performed on anesthetized mice with ketamine (34 mg/kg) and xylazine (12 mg/kg). After tracheostomy, a 18-gauge stainless-steel cannula was inserted into the trachea, and mice were placed on the FlexiVent system (Scireq, Montreal, QC, Canada) for forced oscillatory measurements. Ventilation was maintained at a rate of 150 breaths/min and a tidal volume of 10 mL/kg, with a positive end expiratory pressure of 3.0 cm of H_2_O to prevent alveolar collapse. Total lung capacity (TLC), Snapshot, Quickprime-3, were consecutively performed using the Flexivent system. A TLC perturbation maximally inflates the lungs to a standard pressure of 30 cm H_2_O followed by a breath hold of typically a few seconds to establish a consistent volume history. A single-compartment model of respiratory mechanics was used to assess lung function at 0, 1.5, and 24 h, and airway responses to methacholine (0, 25 and 50 mg/mL) were assessed 24 h after LPS. Total respiratory system resistance (*R*) was determined by snapshot perturbation maneuver. Resistance measurements were made using a 1.25 s, 2.5 Hz volume-driven oscillation applied to the airways by a computer-controlled piston (SnapShot perturbation). Methacholine was aerosolized for 10 s followed by 10 s of ventilation with an ultrasonic nebulizer (Aeroneb; Aerogen), and 25 SnapShot perturbations were performed. The maximum *R* value with a coefficient of determination of 0.9 or greater (as determined by the flexiVent software) was used to determine the dose-response curve. 

### 2.4. Tracheal Segments and LPS Incubation

Mice were exsanguinated by the abdominal aorta under deep anesthesia (ketamine/xylazine at 100 and 20 mg/Kg i.p, resp.). The thorax was opened, and the trachea and lungs were removed *en bloc *as previously described [23]. Tracheal ring segments were dissected, free of connective tissue, at 1 cm above the bifurcation. Two-millimeter-thick segments were incubated in 96 microtiter culture plates containing DMEM medium supplemented with penicillin (100 U/mL) and streptomycin (10 mg/mL) in a humidified atmosphere of 5% CO_2_ and 95% air at 37°C in the absence or presence of LPS (5 *μ*g/mL, *E. coli* serotype 055: B5, Sigma St Louis, MO, USA) or recombinant murine TNF*α* (10 ng/mL, R&D systems) or NaCl solution (0.9%) (Control group) during 90 min. In a parallel set of experiments, the tracheal rings were incubated with monoclonal anti-TNF*α* antibody (5 *μ*g/mL, MP6-XT22). The monoclonal anti-TNF*α*, IgG1 antibody [24], was purified from supernatants of the hybridoma line cells (ATTC), kindly provided by Professor Fernando Cunha (University of Sao Paulo, Brazil), for 30 min before the incubation with LPS.

### 2.5. Removal of Tracheal Epithelium

The tracheal epithelium was removed by inserting a polyethylene tube into its lumen and by gently rubbing it with a corkscrew motion 5 time [25]. The absence of the epithelium was confirmed by histological analysis of tracheal sections, showing contractile response to hypertonic KCl (60 mM). 

### 2.6. Setup of Isolated Ring Trachea and MCh Reactivity

The tracheal contraction analysis was performed using a myograph (Multiwire myograph System 610M, DMT, Aarhus, Denmark). Tracheal rings were suspended with the aid of two steel hooks in an organ bath chamber filled with 10 mL of Krebs-Henseleit buffer-solution (KHS) of the following composition (in mM): NaCl 115, KCl 4.6, CaCl_2_·2H_2_O 2.5, KH_2_PO4 1.2, MgSO_4_·7H_2_O 2.5, NaHCO_3_ 25, and glucose 11. The KHS was continuously gassed (5% CO_2_ and 95% O_2_) and maintained at 37°C. Rings were allowed to equilibrate for 40 min under a tension of 5 mN. During this period, the KHS was replaced every 10 min. Following the equilibrium period, the reference contractile response was assessed by the addition of Krebs-solution containing 60 mM KCl. After washings and return to basal tone, cumulative dose-response curves to MCh were constructed [26]. In brief, tracheal force contractions in response to increasing logarithmic graded doses of MCh added to the organ bath system were recorded through an isometric transducer connected to the PowerLab 4sp system, and data were analyzed using the Chart 3.4 software (Ad Instruments, Australia). 

### 2.7. Statistical Analysis

Data are expressed as mean ± standard error of the mean (S.E.M.). Statistical evaluation of differences between the experimental groups was determined by using unpaired *t*-test when comparing two groups or a two-way analysis of variance, followed by a Bonferroni posttest when comparing more than two groups. All tests were performed with a 4.0 version of the GraphPad InStat software. *P* < 0.05 was considered as significant. *P < 0.05, **P < 0.01, ***P < 0.001.

## 3. Results

### 3.1. In Vivo Measurements of Basal and Methacholine-Induced Respiratory Parameters after Intranasal LPS Administration

**Figure 1:**
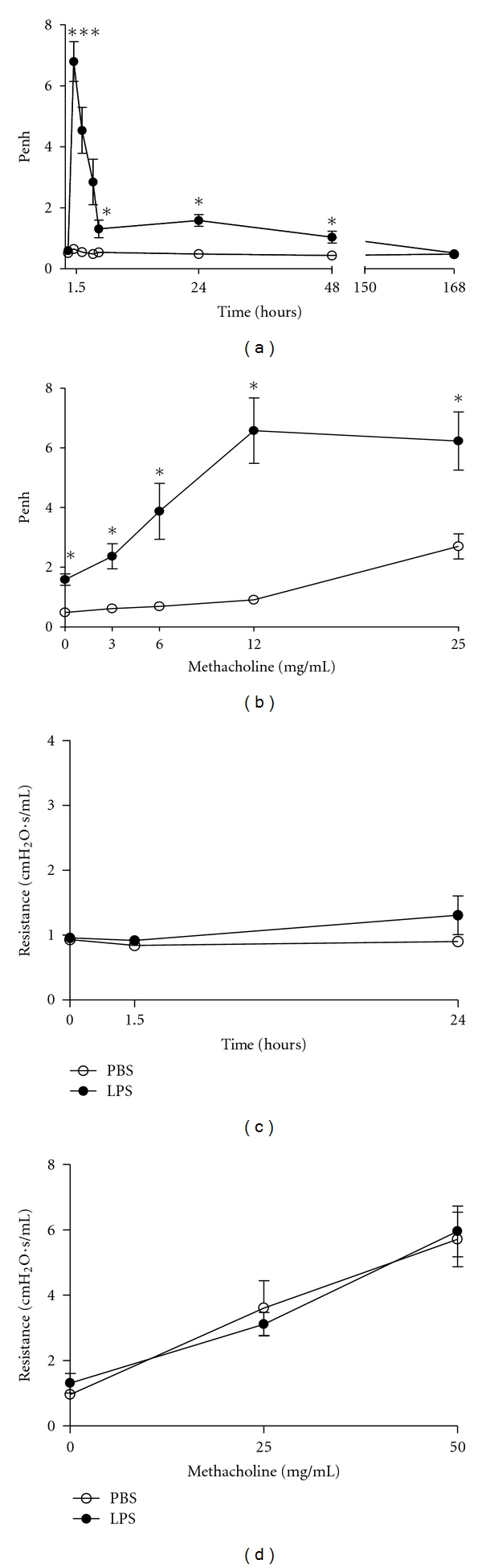
LPS administration increases basal and methacholine-induced respiratory patterns but not airway resistance. Respiratory parameters were determined in C57BL/6 mice by noninvasive (Buxco apparatus) or invasive (FlexiVent apparatus) barometric plethysmography. Penh values or resistance were used as index of the respiratory parameters obtained before (a and c) and after (b and d) sequential delivery of increasing concentrations of methacholine 24 h after instillation of PBS (open circles) or LPS (filled circles). Results are reported as Penh values (a and b) or resistance (c and d). Penh data represent the means ± SEM of five mice per group and resistance data represent the means ± SEM of three to five mice per group (unpaired *t-*test, *P < 0.05, **P < 0.01 as compared to control).

We first determined *in vivo* respiratory pattern by noninvasive plethysmography after LPS administration because this method allows measuring the respiratory pattern of the same animal in different time points. The respiratory pattern was profoundly altered after 1.5 hour of intranasal administration of 20 *μ*g of LPS, decreasing markedly during the following 48 h and more slowly thereafter, reaching basal levels only after 168 hours (7 days) of LPS administration (Figure 1(a)). Intranasal administration of PBS (control group) did not alter the respiratory pattern (Figure 1(a)). These results indicate that LPS alters significantly the basal respiratory pattern during the first 48 hours and markedly 1.5 h after LPS administration. Because the respiratory pattern was still slightly altered after 24 h of LPS, we determined at this time point the airway responses of mice to increasing doses of methacholine. We found that the values of the respiratory pattern increased further and these values were significantly different from PBS group (Figure 1(b)). After 7 days, we could not find any difference between PBS and LPS in the methacholine-evoked respiratory pattern (data not shown). These results are in line with previous results obtained by Noulin et al. [27], in which intranasal LPS administration affected the basal and methacholine-induced respiratory patterns. Because the alterations in the respiratory pattern were so evident with the noninvasive method, we wanted to confirm these results using an invasive method and for this we selected three time points (0 h, 1.5 h, and 24 h) to avoid excessive animal use that this methodology requires. To our surprise, we could not detect any difference when comparing PBS with LPS group in basal (Figure 1(c)) or methacholine-evoked (Figure 1(d)) airway resistance. We conclude that unrestrained noninvasive plethysmography detects changes in airway parameters that are not correlated with airway resistance determined by invasive methodology. Since the *in vivo* results with LPS were conflicting, we sought to dissect the molecular pathways underlying LPS effect on airway smooth muscle contraction using an *in vitro* method with tracheal rings.

### 3.2. Enhancement of Methacholine-Evoked Tracheal Rings Contraction by LPS Is Epithelium and TNF Dependent

**Figure 2:**
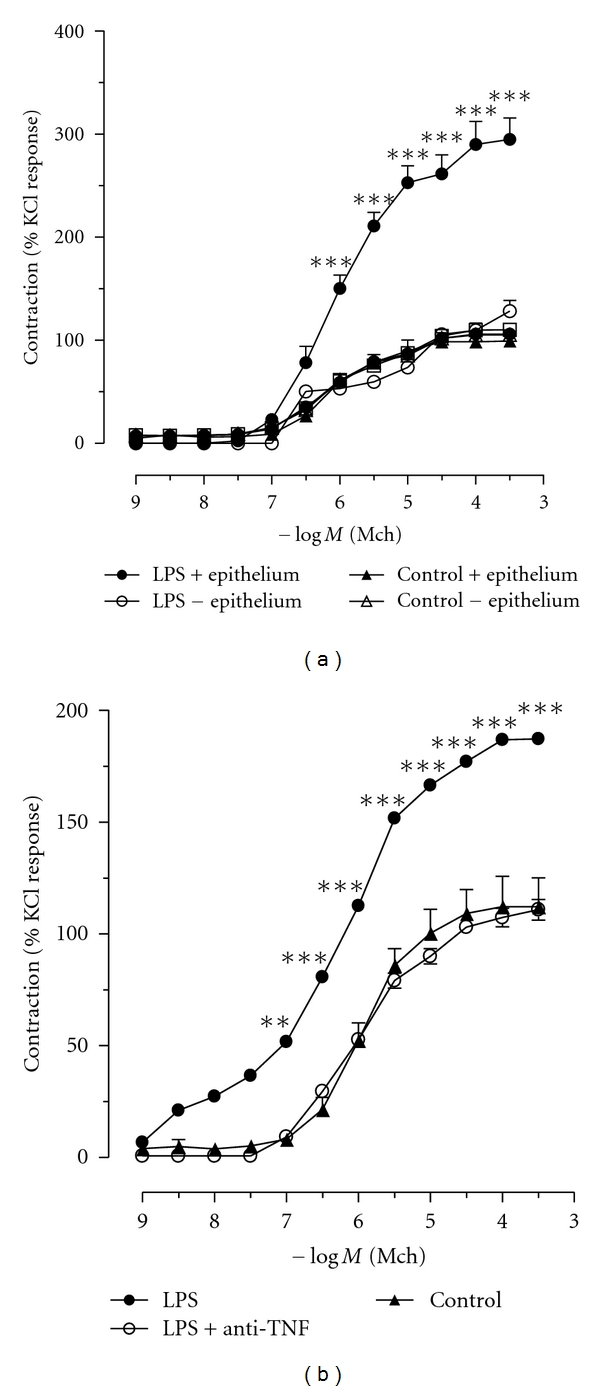
LPS-enhanced methacholine response of isolated trachea depends on intact epithelium and endogenous TNF. (a) Tracheal segments from C57BL/6 mice were incubated with (a) LPS (5 *μ*g/mL-90 min) in the presence (filled circle) or in the absence of epithelium (open circle). Control tissues were obtained by incubating tracheas with saline in the presence (filled triangle) or absence (open triangle) of epithelium (b) LPS (5 *μ*g/mL-90 min) in the presence (filled circle) or in the absence of anti-TNF antibody (open circle). Control tissues were obtained by incubating tracheas with saline in the absence of TNF. All segments used in Figure (b) presented epithelium. The contractile response to increasing concentration of methacholine was recorded using a myograph. Data are expressed as mean ± SEM and are representative of 5–8 experiments (two-way ANOVA with Bonferroni's Multiple Comparison Test. ***P < 0.001; **P < 0.01 as compared to control).

In order to reduce the complexity and the controversy of *in vivo* experiments, we determined the direct/indirect effects of LPS on the airway smooth muscle contraction *in vitro*. Tracheal rings were exposed to LPS (5 *μ*g/mL) for 90 min, washed with PBS, and then the contractile responses to increasing concentrations of methacholine (MCh) were measured.  Figure 2(a) shows that incubation with LPS increased ring contractions evoked by MCh. We did not find any modification in the basal tone of the rings even after 6 h of LPS exposure (data not shown). Because epithelium might participate in the LPS response, we next determined the role of the epithelium on LPS-induced tracheal hyperreactivity. We found that after mechanical removal of the epithelium, tracheal rings no longer showed the enhancement of the MCh-evoked contractile response conferred by LPS exposure (Figure 2(b)). Therefore, LPS most likely acts upon the epithelium in releasing mediators that cause an increased smooth muscle contraction evoked by MCh. In contrast, MCh-evoked contractions in control experiments have the same amplitude with intact or removed epithelium. Since LPS rapidly induces TNF*α* production [27], we asked whether TNF*α* might account for LPS-induced hyperreactivity of the tracheal ring. We determined whether endogenous TNF*α* might mediate the enhanced contractile response evoked by MCh. Thus, we performed experiments in the presence of TNF*α*-neutralizing antibody. As observed in Figure 2(b), TNF*α* neutralization did prevent LPS-induced tracheal hyperreactivity to MCh. Control antibody isotype was used, and the tracheal reactivity to MCh did not differ from control group (data not shown). Therefore, it is likely that LPS activates the production of TNF*α* by the epithelium, which in turn enhances responsiveness of the smooth muscle cells.

### 3.3. TNF*α*-Induced Methacholine-Evoked Tracheal Rings Contraction Is Epithelium Independent

**Figure 3:**
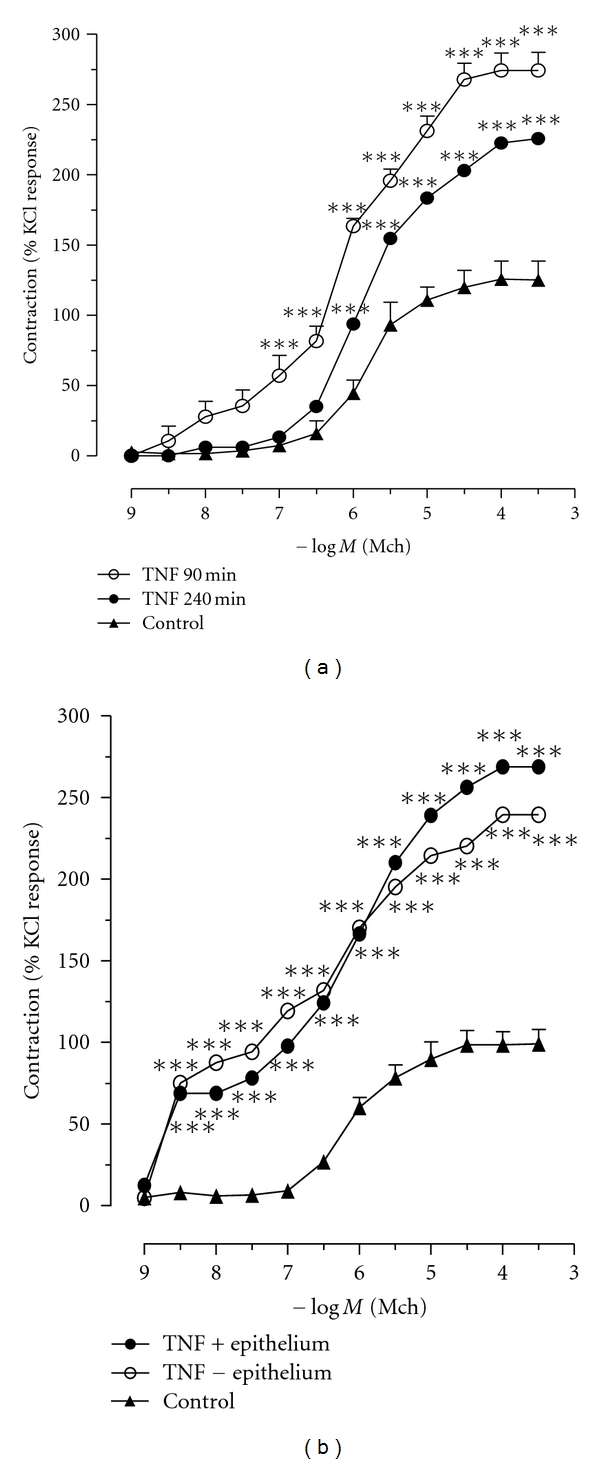
TNF*α* enhances methacholine response of isolated trachea. (a) Tracheal segments from C57BL/6 mice were incubated with TNF*α* (10 ng/mL) for 90 min (open circle) or 240 min (filled circle) in the presence of epithelium. Control tissues were obtained by incubating tracheas in the presence of epithelium with saline (filled triangle); (b) Tracheal segments from C57BL/6 mice were incubated with TNF*α* (10 ng/mL for 90 min) in the presence (filled circle) or in the absence (open circle) of epithelium. Control tissues were obtained by incubating tracheas with saline for 240 min (filled triangle) in the presence of epithelium. The contractile response to increasing concentrations of methacholine was recorded using a myograph. Data are expressed as mean ± SEM and are representative of 4 experiments (two-way ANOVA with Bonferroni's Multiple Comparison Test. ***P < 0.001 as compared to control).

To test the effect of TNF on tracheal contraction, we first determined the effect of recombinant TNF*α* at 1 *μ*g/mL and 10 *μ*g/mL on tracheal rings. The lowest dose did not modify the tracheal reactivity, whereas the preincubation with TNF*α* (10 *μ*g/mL) significantly increased the tracheal ring contractions evoked by MCh that mimicked the LPS-induced hyperreactivity. The effect of TNF was evident at 90 min and slightly reduced after 240 min of incubation with recombinant TNF (Figure 3(a)). In a second step, we tested whether TNF*α*-induced tracheal hyperreactivity to MCh is dependent or independent of the epithelium. We found that TNF*α*-induced hyperreactivity to MCh persisted on tracheal rings devoid of epithelium (Figure 3(b)). 

**Figure 4:**
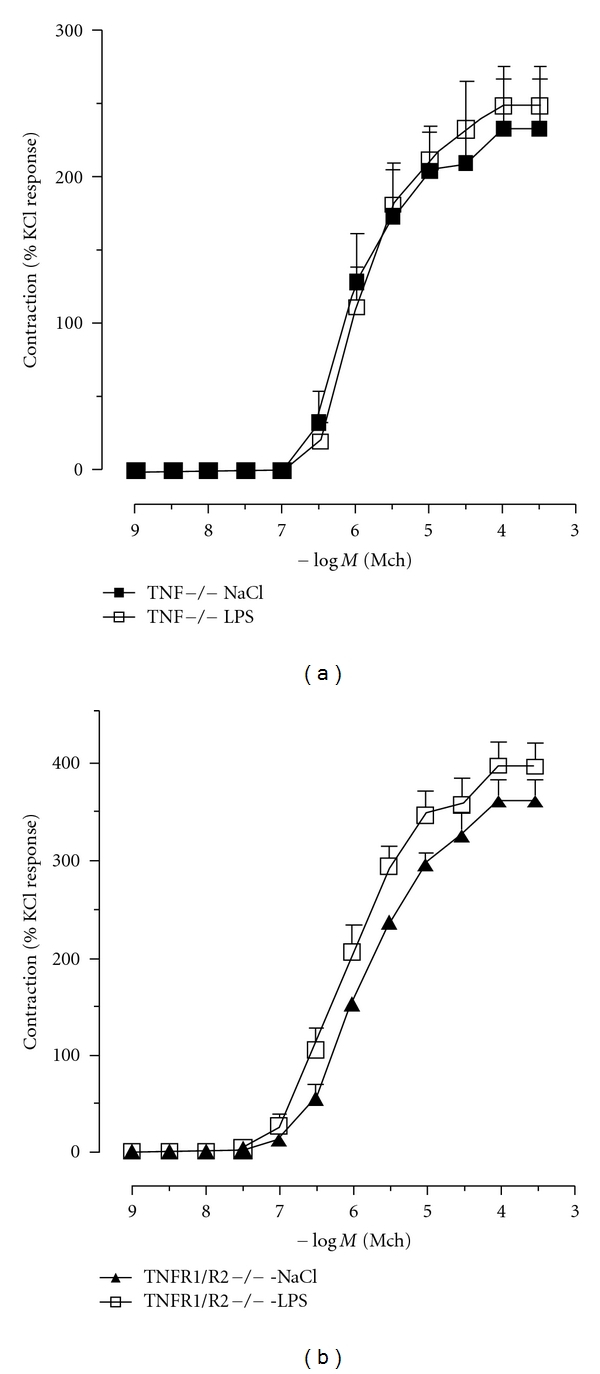
LPS-enhanced methacholine response of isolated trachea depends on the expression of TNF or TNF receptor 1 and 2. Tracheal segments were isolated from WT C57BL/6, TNF^−/−^, or TNFR1R2^−/−^ mice and incubated with LPS (5 *μ*g/mL for 90 min). Control tissues were obtained by incubating tracheas with saline. The contractile response to increasing concentration of methacholine was recorded using a myograph. Data are expressed as mean ± SEM and are representative of 5 experiments (unpaired *t-*test, *P < 0.05, **P < 0.01 as compared to control).

To further confirm a critical role of TNF*α*, we used tracheal rings obtained from mice lacking TNF*α* or its receptors. In line with the data obtained in experiments where TNF*α* was neutralized (Figure 4), LPS failed to induce hyperreactivity in trachea rings isolated from TNF*α*- and TNFR1/R2-deficient mice as evidenced by the responses to increasing doses of MCh (Figure 4). Tracheal rings from TNFR1/R2-deficient mice showed dose-response curves similar to those obtained with wild-type C57B6 mice, with nonsignificant differences in threshold or ED50 (data not shown). However, in these mice, maximal basal response evoked by MCh (control group) was higher than that obtained in WT C57B6 mice (Figure 4). 

### 3.4. LPS-Induced Tracheal Hyperreactivity Is Mediated by Toll-Like Receptor 4 (TLR4)

**Figure 5:**
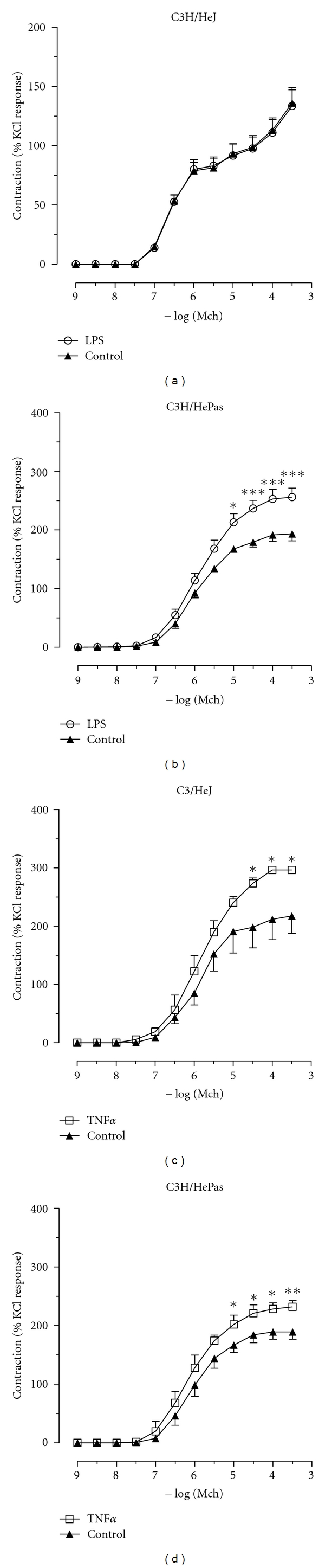
Effect of LPS and TNF*α* on maximal responses evoked by MCh in spontaneously TLR4-deficient C3H/HeJ mice and their counterpart controls, C3H/HePas mice. Tracheal segments were isolated from C3H/HeJ (a and c) and C3H/HePas (b and d) mice and incubated with LPS, 5 *μ*g/mL for 90 min, (a and b) or with TNF*α*, 10 ng/mL for 90 min, (c and d) control tissues were obtained by incubating tracheas with saline. The contractile responses to increasing concentrations of methacholine were recorded using a myograph. Data are expressed as mean ± SEM and are representative of 5 experiments (unpaired *t*-test. *P < 0.05, **P < 0.01, ***P < 0.001 as compared to control).

We previously showed that TLR4 mediates the LPS-induced lung inflammation and changes in respiratory pattern as recorded by noninvasive plethysmography [14]. To investigate the potential role of TLR-4 in LPS-induced hyperreactivity of the trachea, C3H/HeJ mice, a strain with point mutation in TLR-4 receptor, and their counterpart controls C3H/HePas mice were used. We found that the responses to MCh were identical in control and LPS preincubated C3H/HeJ mice (Figure 5(a)), but not in C3H/HePas mice where LPS-induced hyperreactivity to MCh-evoked contraction was significant (Figure 5(c)). In C3H/HeJ irrespective of the MCh concentration used, the tracheal contractions in control and LPS group were similar (Figure 5(a)). Threshold and ED50 were not different and maximum MCh-evoked tensions were 149 ± 7 and 149 ± 8% in control and LPS incubated rings, respectively. The former maximum value is only slightly larger than the values obtained with C57B6 wild-type mice, showing that the absence of hyperreactivity could not result from potentiated control contraction as recorded with mice KO for TNF*α* or TNF receptors. Finally, we determined the response to TNF*α* in these two mouse strains. We found TNF*α*-induced hyperreactivity in C3H/HeJ, TLR4-deficient and in C3H/HePas-, TLR4-sufficient mice (Figures 5(c) and 5(d)). 

## 4. Discussion

This study examined the effect of LPS on contractile airway responses. We found that intranasal LPS administration alters the respiratory pattern and contractile responses to MCh when analyzed by noninvasive (unrestrained plethysmography) but not when determined by invasive method. Our results with noninvasive method confirm previous reports [27, 28]), whereas the lack of significant response observed with invasive method might be related to the gender as it has been shown that only LPS-treated male mice exhibit an evident increase of MCh-evoked airway resistance [29]. Because determinations of pulmonary function by noninvasive versus invasive methods are still very controversial [30, 31], we extended our findings by showing that murine tracheal rings exposed to LPS display an enhanced response to MCh. In a murine model of asthma, it was shown that noninvasive *in vivo* determination of airway reactivity to methacholine correlated with smooth muscle contraction of tracheal rings [32]. The responses of tracheal rings are well established with a clear role of TNF in modulating hyperreactivity, as reviewed by Amrani et al. [33]. Indeed, we also showed that LPS-induced hyperreactivity is dependent upon tracheal epithelium integrity, endogenous TNF*α* production, and TNF receptors. 

Cholinergic airway hyperresponsiveness has been reported during lung neutrophilic inflammation [8, 34]. Lung inflammation has been considered the main cause of bronchial hyperresponsiveness [35] and LPS inhalation causes *in vivo* airway hyperresponsiveness involving lung inflammation [36]. Nevertheless, a causal link between the augmented airway responsiveness and lung inflammation is still open for discussion [36], demanding additional studies. 

The present data demonstrated that LPS augments the* in vivo and in vitro *response of the airways to cholinergic agonist. Of note in our *in vitro* experiments is the fact that LPS was removed from the medium before the MCh-reactivity studies, and this argues against a direct effect of LPS on tracheal hyperreactivity *per se.* Our findings are in line with previous studies that showed LPS-induced hyperreactivity *in vivo* [28, 34, 37]. However, we extended our findings by determining whether LPS-induced tracheal hyperreactivity was dependent upon epithelium integrity. Epithelial removal of the trachea prior to incubation with LPS blunted the hyperreactivity to MCh, indicating that LPS stimulates tracheal epithelium that, in turn, mediates hyperreactivity to MCh. It is now well documented that the airway epithelium is more than a simple diffusion and protective barrier is now [38–40]. Indeed, it has been shown the epithelial cells release various mediators that potentially can modulate a large number of targets within the airways including airway smooth muscle constriction or relaxation [41]. Balzary and Cocks [16] carefully studied the signaling pathways of the TLR4-mediated LPS-induced relaxation of precontracted mouse-isolated trachea and found it was mediated by the release of prostaglandin PGE2. However, Bachar et al. [42] showed that TNF*α* attenuates the smooth muscle relaxation induced by prostaglandins. Overall, the data suggest a scenario in which TNF-*α* and other mediator released by the epithelium upon LPS exposure might be responsible for cholinergic hyperreactivity [16]. Experiments show that not only TNF*α* mimicked the effect of LPS but also that the monoclonal anti-TNF*α* antibody suppressed the LPS-induced hyperreactivity to MCh, thus promoting TNF*α* as a major mediator involved in LPS-induced airway hyperreactivity. LPS induces the release of a variety of inflammatory mediators [43] including TNF*α*. The latter is released by airway smooth muscle [44] and exerts an important role on the airway contractile response [45]. TNF*α* is released after the interaction of LPS with the TLR4 [44] and is also involved with *in vivo* airway constriction [46], including increased airway responsiveness in nonasthmatic human subjects [47]. Indeed in our experiments, TNF*α*-induced tracheal hyperreactivity to MCh was independent of airway epithelium. Altogether, our data support the following scenario: LPS-epithelium interaction releases TNF*α*; TNF*α* interacts with smooth muscle cells and enhances MCh-evoked contraction. MCh-evoked contraction by TNF*α* can involve multiple targets such as signal transduction, calcium stores, and contractile machinery [33], which need to be appropriately addressed. 

Since it is established that LPS responses are mediated by broadly expressed TLR4, experiments were performed with the mutant TLR4-deficient C3H/HeJ mice. In these mice, the LPS induced hyperreactivity to MCh was abolished, whereas it was maintained in their counterpart controls, C3H/HePas mice as it was for TNF*α* in both mice strain. TLR4 expression has been described in the tracheal muscle layer [48, 49] (Bachar et al., 2004; Shan et al., 2006). In addition, Hammad et al. [50] showed preferential TLR4 immunolabelling in epithelial cells and macrophages. The current study showing both TLR4 and tracheal epithelium requirement together with the TLR4-LPS interaction suggests that the LPS-induced hyperreactivity involves epithelial cells expressing TLR4. The TNF effect on airway smooth muscles could be via the activation of TNFR1/TNFR2 as shown by Chen et al. [51]. In an attempt to examine the potential role of TNF*α* and its receptors, TNF*α* and TNF receptors KO mice were used. The fact that LPS failed to increase the response to MCh in these mice suggests a key role of TNF*α* and its receptors in mediating hyperreactivity. Moreover, the use of neutralizing antibodies to TNF*α* directly demonstrated the pivotal role of TNF*α* in the LPS-induced hyperreactivity. 

In conclusion, we report that LPS activates TLR4 on epithelial cells of the trachea and TNF-*α* via TNFR1/TNFR2 appears to be involved in the LPS-induced hyperreactivity to MCh of tracheal smooth muscle cells.

## Abbreviations

LPS: Lipopolysaccharide
MCh: Methacholine
MyD88: Myeloid Differentiation Factor 88
TLR: Toll-like receptor
TNF*α*: Tumor necrosis factor-*α*.
